# Implementing Peer Tutoring for the Development of Empathy in Nursing Education

**DOI:** 10.17533/udea.iee.v39n2e07

**Published:** 2021-06-15

**Authors:** David Durán Gisbert, Anabel Vázquez Rivas

**Affiliations:** 1 Educational Psycologist. Ph.D. Associate Professor, Universitat Autònoma de Barcelona, Spain. Email: david.duran@uab.cat Universitat Autónoma de Barcelona Universitat Autònoma de Barcelona Spain david.duran@uab.cat; 2 Nurse. Ph.D. Adjunct Professor, Universitat de Barcelona, Spain. Email: anabelvazquezrivas@ub.edu Universitat de Barcelona Universitat de Barcelona Spain anabelvazquezrivas@ub.edu

**Keywords:** education, nursing, empathy, learning, peer group, psychology, students, nursing., educación en enfermería, empatía, aprendizaje, grupo paritario, psicología, estudiantes de enfermería., educação em enfermagem, empatia, aprendizagem, grupo associado, psicologia, estudantes de enfermagem.

## Abstract

**Objective.:**

This research sets out the effects of a training method based on peer tutoring, aimed at developing empathy among nursing students at the University of Barcelona (Spain).

**Methods.:**

After initial training, students are matched in pairs with similar level of empathy, exchanging the role of tutor and tutee in every session, during 12 sessions. Before the session, the tutor prepares the activities to work with his or her tutee, following this structure: exploration of prior knowledge, explanation, practical activities, feedback, and reflection. Jefferson Scale of Empathy was administered as pre-test and post-test to 76 nursing students, 40 in the intervention group, and 36 in the comparison group. Following a mixed-methods sequential explanatory design, a quantitative study (a quasi-experimental design with a comparison group) was combined with a qualitative study (interaction analysis of the 12 videotaped sessions).

**Results.:**

The results revealed statistically significant improvements in empathy in the intervention group. Subsequent analysis of the peer tutoring interaction highlighted the specific actions that had resulted in these improvements and generated a context in which the tutee was able to understand complex concepts, while enabling both students to participate, reflect and discuss.

**Conclusion.:**

Peer tutoring is an effective method for the development of empathy in nursing students.

## Resumen

**Objetivo.:**

Esta investigación evaluó los efectos de un método de formación basado en la tutoría entre iguales, destinado al desarrollo de la empatía en estudiantes de enfermería de la universidad de Barcelona (España).

**Métodos.:**

Después de la formación inicial, los estudiantes son emparejados con compañeros con un nivel similar de empatía, intercambiando el papel de tutor y tutorado en cada sesión, durante 12 sesiones. Antes de cada sesión, el tutor prepara las actividades para trabajar con su tutorado, siguiendo esta estructura: exploración de conocimientos previos, explicación, actividades prácticas, retroalimentación y reflexión. Se administró la Escala de Empatía de Jefferson, en forma de pre y postest a 76 estudiantes de enfermería: 40 en el grupo de intervención y 36 en el grupo de comparación. Siguiendo el método mixto de diseño explicativo secuencial, se combinó un estudio cuantitativo (un diseño cuasiexperimental con grupo de comparación) con un estudio cualitativo (análisis de interacción de las 12 sesiones grabadas en video).

**Resultados.:**

Los resultados revelaron mejoras estadísticamente significativas en la empatía en el grupo de intervención. El análisis posterior de la interacción en las parejas de tutoría entre iguales puso de relieve las acciones específicas que habían dado lugar a estas mejoras y generando un contexto en el que el tutorado era capaz de entender conceptos complejos, al tiempo que permitía a ambos estudiantes participar, reflexionar y discutir**.**

**Conclusión.:**

La tutoría entre iguales es un método eficaz para el desarrollo de la empatía en los estudiantes de enfermería.

## Resumo

**Objetivo.:**

Esta pesquisa avaliou os efeitos de um método de treinamento baseado em tutoria entre pares, com o objetivo de desenvolver empatia em estudantes de enfermagem da Universidade de Barcelona (Espanha).

**Métodos.:**

Após a formação inicial, os alunos são colocados em pares com pares com um nível de empatia semelhante, trocando o papel de tutor e estudante em cada sessão, durante 12 sessões. Antes de cada sessão, o tutor prepara as atividades para trabalhar com o seu estudante, seguindo esta estrutura: exploração dos conhecimentos anteriores, explicação, atividades práticas, feedback e reflexão. A Escala de Empatia de Jefferson foi administrada em forma de pré e pós-teste a 76 estudantes de enfermagem: 40 no grupo de intervenção e 36 no grupo de comparação. Seguindo o método misto de desenho explicativo sequencial, um estudo quantitativo (um desenho quase experimental com um grupo de comparação) foi combinado com um estudo qualitativo (análise de interação das 12 sessões gravadas em vídeo).

**Resultados.:**

Os resultados revelaram melhorias estatisticamente significativas na empatia no grupo de intervenção. A análise subsequente da interação nos pares de tutoria de pares destacou as ações específicas que levaram a essas melhorias e gerou um contexto em que o estudante foi capaz de compreender conceitos complexos, permitindo que ambos os alunos participassem, refletissem e discutissem.

**Conclusão.:**

A tutoria entre pares é um método eficaz para desenvolver empatia em estudantes de enfermagem.

## Introduction

Empathy is a complex, cross-dimensional interpersonal skill. It is particularly essential for nursing professionals to help them care for patients and their families in a holistic manner and establish a therapeutic relationship.(1) Despite the difficulty to define the concept, many studies coincide in describing empathy as the skill to put oneself in the patient’s shoes, while retaining one’s own point of view, and ensuring that the patient knows that his/her point of view has been understood.(2) In the sphere of healthcare, considering empathy as a multi-dimensional element, Hojat(3) defines it as a predominantly cognitive attribute (rather than an emotional one), involving the comprehension (rather than the feeling) of patients’ experiences, concerns, and perspectives, combined with the skill to convey this comprehension to them. Empathy therefore constitutes a basic element in the therapeutic relationship between the patient and the professional healthcare, beneficial both for patients and their families, as well as for nursing staff themselves and the health institution as a whole. Studies have demonstrated that patients that are cared for by highly empathetic nursing staff show reduced levels of anxiety and depression, lower hostility towards healthcare professionals and increased degree of satisfaction with the care received.(4)

For nursing professionals, the contact with the patient’s suffering, tackling difficult and stressful emotional situations, overwork and lack of social support make them vulnerable to burnout and anxiety.(5) Furthermore, if the nursing staff has institutional and social support, they are less likely to be susceptible to burnout, anxiety, depression, and hostility.(6) The complex nature of empathy as a multi-dimensional concept makes it difficult to gauge.(7) Most studies measure it using quantitative methods such as the Jefferson Scale, given its wide acceptation among the scientific community, its extensive use in different health specialities, and its translation into different languages.(3)

Even though it is widely recognised that empathy should be further developed in healthcare professionals, during initial training there are few deliberate education opportunities with effective methods to develop empathy.(8) However, empathy can be developed through different types of activities, based on active methodologies -for example, experiential activities using real or simulated patients,(9) or peer tutoring.(10) In a formal training context, peer tutoring is described as a peer learning method based on the creation of pairs, with an asymmetric relationship (the role of tutor and tutee and their respective tasks) and a common and shared objective, which is the acquisition or improvement of some curricular competence, through structured interactions planned by the teacher.(11) Peer tutoring meta-analyses present numerous positive effects,(12,13) as well as being a potentially useful and effective methodology to improve cognitive, social, and communicative skills.(14) Peer tutoring may involve fixed roles (the tutor and tutee always play the same role), or reciprocal ones (the tutor and tutee interchange their roles). (11) In this study, a reciprocal role type is proposed to give each student the chance to perform both roles.

In the field of nursing education, peer learning is presented as an educational model highly suitable for clinical placements, due to the increasing number of students and a limited number of preceptors.(15) Research on recent practices that use peer learning in clinical practice education shows positive effects on nursing students’ self-efficacy(16) and professional competence.(17) Irvine *et al.*(18) carried out a review of 29 studies, between the years 1990 and 2017, and reported benefits of peer teaching in creating a safe supportive learning environment, learners viewing near-peer teachers as effective role models, and increased confidence experienced by learner and teacher. The reviewed studies mainly focused on cognition with little emphasis on metacognition or affective behaviours, and lacked training provided to tutors or peer teachers. The authors concluded that it is imperative that faculty embed near-peer teaching into the curriculum, but more studies are needed in order to provide definitive evidence supporting this pedagogical approach and a theoretical framework for its implementation, particularly from educational psychology. Given the importance of empathy as an integral part of the skillset required for nursing staff, and the need to find ways of developing it during the initial training period of these professionals, the objective of this study is to design a pedagogic project based on peer tutoring and explore its potential to develop empathy both in terms of positive aspects and areas of improvement, with a view to incorporating it into initial nursing staff training.

## Methods

This objective is summarised as a hypothesis and three questions. The hypothesis is that the students participating in the project will self-report a greater degree of empathy, obtaining statistically substantial differences in the Jefferson Scale (Spanish version JSPE-S questionnaire,(19) between the pretest and posttest, while the comparison group is not expected to reveal any statistically significant changes. In order to explain the possible quantitative changes, the qualitative work, focused on analysing the process, aims to reveal which elements in the interaction contribute to the development of empathy in students. To do this, three questions are formulated: (1) In the first part of the peer tutoring session, where the tutor explains basic concepts to the student, what actions of the pair help or hinder the development of conceptual knowledge of empathy and why?; (2) In the second part of the peer tutoring session, where the different activities designed are put into practice, what actions of the pair help or hinder the emotional development of empathy and why?, and (3) In the final part of the peer tutoring session, where there is joint and individual reflection on what has been learned and undertaken, which aspects help raise awareness of the development of empathy itself and why?

Research Design. This research opts for a mixed-methods sequential explanatory design,(20) combining a quantitative study (a quasi-experimental design with a comparison group); and a qualitative study (an analysis of the data extracted from the interactions between each pair of students) to explain the quantitative changes detected.(21)

Sample. The sample consists of 76 third-grade nursing students in clinical internships at the University of Barcelona. To preserve the ecological validity of the study, two groups of students from equivalent degrees, consisting of 40 and 36 students, were randomly assigned as comparison or intervention group. The 40-student class became the intervention group and the 36-student class was set as comparison group.

Intervention: Training Programme and Procedure. Prior to the sessions, an initial training session is held, explaining the programme and what peer tutoring entails, and students are administered an initial assessment using the JSPE-S test. Based on the scores, the pairs are formed, having the student with the highest empathy score paired with the student with the second highest score, and so on consecutively. Therefore, a similar level of skill is guaranteed among the two members of the pair, which -as indicated by extant literature- is essential for the use of reciprocal peer tutoring, in which the roles of tutor and tutee alternate. (11) The programme is designed based on the systematic review of actions favouring empathy development and consists of six one-hour sessions of reciprocal peer tutoring in two sessions per week.(2,22) Working in pairs, the students are given two different dossiers (one for student A and one for student B) specifying what needs to be done throughout the session guided by an activity clock. This dossier contains the materials (in the form of text or audio-visual resources) that tutors need to prepare prior to the session. Student A acts as the tutor during the first hour, and B during the second hour, to give them both the chance to perform each role.

Sessions are structured by means of an activity clock: graphical structure that serves as a guideline so that students learn a work routine in each session and can be increasingly more autonomous when controlling each activity’s timeline. Each session has the following time frames: ‘Prior Training’ (5 minutes): the tutor explores what his/her tutee knows about the subject before the session begins; ‘Explanation’ (10 minutes): tutor uses the teaching material he/she has prepared to explain what he/she knows to the tutee; ‘Practical Session’ (30 minutes): tutor guides the activities using role play, visualisation and video analysis, and written activities; ‘Feedback between Pairs’ (5 minutes); and ‘Reflection and End of Session’ (10 minutes): the pairs answer the Self-assessment questions from the dossiers. An example of a practical activity is to share a personal experience of emotional significance that created an internal conflict. The tutor explains the experience while the tutee listens to him/her and asks questions if necessary. Then the tutee must explain the situation as if it had happened to him/her. This enables the tutee to perform an activity of reflection based on empathy towards the partner. In the case of videos and role plays, several aspects that are essential for building an empathic therapeutic relationship are analysed, such as appropriate use of verbal and non-verbal communication, developing a good relationship or understanding, and the professional’s implication with the patient.(23) The randomly assigned comparison group is provided with the same learning content, not through peer tutoring but in a teacher-centred explanation for the whole group of students.

Instruments. To quantitatively measure empathy the Spanish version of the Jefferson Scale of Physician Empathy for Healthcare Science Students - JSPE-S- (19) was used (internal consistency of 0.74). This self-questionnaire is comprised of 20 items and a Likert scale of seven categories, where 1 means completely disagree and 7 completely agree. A high score on the JSPE-S suggests a higher degree of self-perceived empathy. The theoretical structure of the questionnaire is based on 3 dimensions: *perspective taking* (point of view that makes the professional unbiased when actively listening to the patient’s concerns and offering empathic responses); *compassionate care or treatment* (the human connection based on care between patient and healthcare professional); and *skill to put oneself in the patient’s shoes* (ability to perceive and understand others’ feelings, entering into the others’ subjective world).

Concerning the qualitive study, to analyse the pair interaction during the project sessions and answer the first three research questions, a category system based on extant literature but situated *ad hoc* was created. The procedure was performed as follows. After recording all the sessions (240 hours), an initial observer created the category system analysing 25% of total time recorded (60 hours). To verify reliability and validity, two previously trained researchers individually coded these videos. The level of coincidence between them reached a value of 0.8 in Pearson coefficient, which indicates that the category system is reliable. The category system is comprised of 16 dimensions, which are in turn subdivided into factors evaluated with a binary answer (Yes/No). The evaluation of each factor is gradual and cumulative, that is, the higher the factor evaluated with a Yes, the better the student’s preparation and explanation offered to the tutee. The dimensions and factors drawn up to analyse the degree of empathy are split into the 3 segments that make up the peer tutoring session that will be analysed: conceptual approximation, comprised of 8 dimensions and 30 factors; the practice, comprised of 5 dimensions and 19 factors; and finally, the reflection, comprised of 3 dimensions and 13 factors.

Data analysis. For the quantitative data, we opted for analysis of variance (ANOVA) for repeated measures, with the purpose of studying the variation over time of both groups regarding the dimensions studied (perspective taking, compassionate care and putting oneself in the patient’s position). Qualitative data were gathered by means of video recording the 6 peer tutoring sessions from 10 pairs -20 students-. Video tapes were analysed by means of the Atlas.Ti software.

Ethical Issues. This study does not involve any conflict of interests or ethical conflict, and all students’ names were anonymised to ensure confidentiality. Explicit consent from each of the students was required and they signed a document to authorise data collection for research purposes in an anonymous and voluntary manner.

## Results

### Quasi-experimental Study Results

First, the homogeneity of both groups was analysed by means of Student’s t-test for independent samples, comparing the different pretest scores between intervention and comparison group, presented in Table 1.

**Table 1:** Scores by student *t* test for independent samples of each dimension between Comparison and intervention groups pre-test

Dimensions	*t*	*gl*	*p-value (2-tailed)*	*Difference between means*	*Typical error of the difference*
Perspective taking	2.129	74	0.037	3.164	1.486
Compassionate care	2.587	74	0.012	2.786	1.077
Putting oneself in the patient’s shoes	0.661	74	0.511	0.389	0.589

The results indicate that, except for the “Putting oneself in the patient’s shoes” score, there are statistically significant differences for both the dimensions “Perspective taking” and “Compassionate care”. This means that groups are not homogeneous because they are part of different pre-test situations, which is not a problem because the study aimed to analyse the variation of different groups even though their initial scores are different. The results obtained with ANOVA regarding the “Perspective taking” dimension in the pre-test-/post-test from the intervention group and the comparison group are presented in Table 2.

The results obtained reveal statistically significant differences between intervention and comparison groups (*p*=0.001). There is an increase in intervention group scores, unlike the non-variation in comparison group. In relation to the dimension “Compassionate care”, the scores variation over time is statistically different between both groups (*p*=0.004). There is an increase in the intervention group scores, but not in the comparison group scores. As regards “Putting oneself in the patient’s shoes” dimension, there are no differences over time between both groups (*p*=0.432). Taking the total scores of the tests, results show that the variation over time is statistically significant between both groups (*p*<0.0005); the total score decreased in comparison group and increased significantly in intervention group.

These results show that both groups have different pretest scores, with a higher mean in the comparison group (119.14) than in the intervention group (112.8). However, the variation over time of both groups is different; intervention group obtains a higher mean in the posttest (118.58), with an increase of more than 5.78 points from the pretest, while the comparison group shows a lower posttest mean (116.08) with a decrease of 3.06 points from the pretest.

**Table 2:** Evolution of dimensions of the Scale of Physician Empathy for Healthcare Science Students over time in the comparison and intervention groups

Dimension		Pre-test	Pre-test	Post-test	Post-test
Perspective Taking*	*n*	mean	S.D.	mean	S.D.
Comparison Group	36	60.89	6.02	60.69	6.38
Intervention Group	40	57.73	6.84	62.30	5.36
Compassionate Care†					
Comparison group	36	43.61	4.795	41.28	4.82
Intervention group	40	40.82	4.590	41.98	4.95
Putting oneself in the patient’s shoes‡					
Comparison Group	36	43.61	4.795	41.28	4.82
Intervention Group	40	40.82	4.590	41.98	4.95
Total scale					
Comparison Group	36	119.14	10.81	116.08	11.09
Intervention Group	40	112.80	11.33	118.58	10.59

Time*group interactions (Huynh-Feldt correction): *: F= 1.91; *p*=0.001; †: F= 8.675; *p*=0.004; (‡): F= 0.623; *p*=0.432; §: F= 15.350; *p*<0.005

### Results of the Interaction Analysis using the Dimensions System

The category system designed to analyse the interaction of the video-taped pairs has 16 dimensions, subdivided into factors that are evaluated using a dichotomous response (Yes/No). The category system is divided into 3 segments that configure the peer tutoring session: the conceptual approach (with 8 dimensions and 30 factors); the practical session (5 dimensions and 19 factors); and finally, the reflection (3 dimensions and 13 factors). For each factor, frequencies and percentages are obtained. Results are presented following the three questions.

#### A) In the conceptual approach segment, which actions in the pair help or hinder the development of the conceptual understanding of empathy?

To answer the first question, the first 15 minutes of all conceptual understanding sessions were analysed (‘Prior Training’ and ‘Explanatory Part’), assessing the pair interaction based on the first eight dimensions created for the analysis. Results are summarized in Table 3. 

**Table 3:** Results of Conceptual Approach Segment (20 pairs of students, 12 sessions)

Dimension / Factors	f	%
1. Preparation of sessions		
1.1 The tutor did not bring the materials	1	1.2
1.2 The tutor brought the materials	56	70
1.3 The tutor brought the materials with underlining	14	17.5
1.4 The tutor brought the materials with underlining and notes on the underlined parts	9	11.2
2. Use of materials during the session		
2.1 The tutor used the extra material provided such as further information	29	36.2
2.2 The tutor created synthesis material (charts, graphs, material in notebooks or paper)	50	62.5
2.3 The tutor created teaching material (additional material such as photos, other documents or resources)	1	1.2
3. Conceptual understanding by the tutor		
3.1 Incorrect comprehension of the concepts	1	1.2
3.2 Correct comprehension of the concepts but in a literal way	5	6.2
3.3 Correct and appropriate comprehension (using own words) of the concepts without using examples	14	17.5
3.4 Correct and appropriate comprehension using examples	60	75
4. Action taken by the tutor to detect tutee’s prior knowledge		
4.1 No questions asked to detect prior knowledge	5	6.2
4.2 The tutor asked the student without subsequently giving an answer	15	18.7
4.3 The tutor asked questions and provided answers	3	3.7
4.4 The tutor asked questions and gave feedback to the tutor	16	20
4.5 The tutor asked questions, assessed the response and helped prompt the tutee’s prior knowledge (giving clues, examples)	41	51.2
5. Building on knowledge through pedagogical guidance		
5.1 The tutor transmitted information	41	51.2
5.2 The tutor used the tutee’s prior knowledge and improved on it	30	37.5
5.3 The tutor recognised the tutee’s prior knowledge and together they built on the framework/ idea/ concept	9	11.2
5.4 The tutor and the tutee built new knowledge based on their prior knowledge	0	0
6. Tutor’s verification of tutees' knowledge		
6.1 No questions asked on understanding	22	27.5
6.2 The tutee was the one asking questions and the tutor simply answered them	28	35
6.3 Both tutor and tutee asked questions on their understanding	15	18.7
6.4 The tutor asked questions on the tutee’s understanding	15	18.7
7. Guiding the interaction		
7.1 The tutor did not guide the interaction	5	6.2
7.2 The tutor guided the interaction during the conversation but did not anticipate the structure of the activity during the session	54	67.5
7.3 The tutor guided the interaction, anticipated the structure of the activity during the session, and guided the conversation	13	16.2
7.4 The tutor guided the interaction by providing guidelines on the activity during the session by recapitulating and concluding different blocks	8	10
8. The tutor stimulates and maintains the tutee’s interest		
8.1 The tutor did not stimulate any interest in the tutee	3	3.7
8.2 The tutor stimulated the interest of the tutee in the task	11	13.7
8.3 The tutor stimulated the interest of the tutee and maintained it	66	82.5

Based on these results, it was considered that students were able to understand not only the concept but also what being empathetic and establishing a therapeutic relationship entails. Furthermore, tutors were able to formulate questions, assess the answers, and help prompt the tutees’ prior knowledge (by giving clues or examples). It was also considered that they were able to synthesise and tell information to their peers in their own words -which indicated that their understanding had been fully interiorised. However, it seems that some actions from the tutors have room for improvement, especially those related to stimulating their tutee’s prior knowledge, avoiding transmission of information without rebuilding it with their tutees or checking the progressive understanding of the concepts, and guiding the session.

#### B) In the practical activity, which of the pair’s actions help or hinder the emotional development of empathy?

The following 30 minutes of all practical sessions were analysed by examining pair interaction, based on the 5 dimensions created for the analysis. The results are shown in Table 4.

**Table 4:** Results of practical session (20 pairs of students, 12 sessions)

Dimension / Factors	f	%
9. Tutor’s involvement in the practical activity		
9.1 The tutor showed little collaboration and prevented the practical activity from being performed correctly	0	0
9.2 The tutor showed little collaboration and made it difficult to perform the practical activity	1	1.2
9.3 The tutor showed little collaboration but completed the practical activity together with the tutee	2	2.5
9.4 The tutor collaborated when the tutee asked for help, and completed the practical activity correctly	8	10
9.5 The tutor collaborated during the practical activity, helping where the tutee required, and allowing the practical activity to be performed correctly	69	86.2
10. Helpfulness: explanation		
10.1 The tutor did not explain the concepts	6	7.5
10.2 The tutor provided explanations when the tutee asked for them	17	21.2
10.3 The tutor provided explanations without the tutee asking for them	57	71.2
11. Helpfulness: questions		
11.1 No interrogation or questions	17	21.2
11.2 Only the tutee asked questions	14	17.5
11.3 The tutor asked questions to the tutee	2	2.5
11.4 The tutor asked questions to the tutee and provided feedback	47	58.7
12. Guidance provided in practical activity		
12.1 The tutee explained the activity to the tutor	4	5
12.2 The tutor did not explain the activity, both tutee and tutor waited for the teacher to explain the activity	58	72.5
12.3 The tutor explained the activity to the tutee, but the tutor did not provide guidance during the activity	5	6.2
12.4 The tutor explained the activity to be done to the tutee and guided the activity considering the objectives proposed in the dossier	13	16.2
13. Objectives specified in the dossier		
13.1 The tutor did not consider the objectives that needed to be reached	2	2.5
13.2 The tutor considered some of the objectives that needed to be reached and the tutee reached them	18	22.5
13.3 The tutor considered the objectives that needed to be reached and the tutee reached them	60	75

It seems that the tutors did consider the objectives of the activity to help the tutees meet them. Tutors also prompted their tutees by asking questions and offering feedback, and generally collaborated throughout the practical session by helping their tutees.

#### C) In the segment of reflection, which elements favoured their awareness on empathy development itself?

The final 15 minutes, defined as the ‘reflection segment’ (feedback plus reflection) were analysed by examining pair interaction, based on the three final dimensions designed. The results, summarized in Table 5, show that students were able to create environments, generate time for reflection and expression, and actively listen to the respective points of view, which are all essential for the practice of empathy.

**Table 5:** Results on Reflection Segment (20 pairs of students, 12 sessions)

Dimension /Factors	F	%
14. Mutual understanding of the questions on reflection		
14.1 Tutee responded to questions on reflection and tutor gave his/her point of view, but no common reflection was made	2	2.5
14.2 Tutor reformulated questions to the tutee to understand his/her point of view and/or to delve into the answers given by the tutee	10	12.5
14.3 Tutor and tutee listened to and understood each other and gave each other time to think and explain their point of view	68	85
15. Reflection on meaning of concepts		
15.1 No assimilation of concepts was shown	2	2.5
15.2 Tutor and tutee understood and discussed the concepts following the conceptual approach and practical activity	15	18.7
15.3 Tutor and tutee understood and discussed the concepts learnt and justified them or gave examples	45	56.2
15.4 Tutor and tutee were able to reconstruct the meaning of the concept	12	15
15.5 Tutor and tutee were able to construct the meaning of the concept by changing the initial ideas	6	7.5
16. Self-assessment as a part of reflection		
16.1 No self-assessment was carried out	30	37.5
16.2 Self-assessment was carried out individually	12	15
16.3 Tutor assessed tutee	1	1.2
16.4 Tutee assessed tutor	7	8.7
16.5 Tutor and tutee assessed each other mutually	30	37.5

But not all the tutors’ actions are in good direction. Results indicate that their reflections on the meaning of the concepts were limited to discussion, indicating that it was correct but superficial. Students’ self-assessment could also be improved to include mutual assessment more frequently.

## Discussion

Given the need to help nursing students develop their empathy, a peer-tutoring intervention has been designed to generate favourable social exchange spaces. The results of the quasi-experimental study show statistically significant improvements in the intervention group which did not occur in the comparison group, especially in the “Perspective Taking” and “Compassionate Care” dimensions. This suggests that this peer tutoring intervention can be effective in promoting social experiences that enhance empathy. The result reinforces the idea that empathy, as a complex social skill, can be developed through teaching methods based on cooperation.(10)

The analysis of pair interaction in the peer tutoring sessions that consist of three segments -conceptual approach, practical activity and self-reflection- can help us better account for the quantitative results. The reported improvement in the “Perspective taking” and “Compassionate Care” dimensions may be due to the creation of an environment that enabled students (in both roles, tutors and tutees) to understand complex concepts, fostered a high level of participation, and provided opportunities for joint reflection and discussion. It seems that peer tutoring was effective given that tutors developed their conceptual learning of empathy by means of preparing materials for the session and using them to explain and question their tutees, which is known as learning by teaching.(24) However, results could probably be better in all dimensions, provided that tutors are encouraged to explain in their own words, adjust the explanations to the interest and characteristics of their tutees and ask and answer deep questions.(25) Nevertheless, it seems that tutees also increased their learning through the help received, which was personalised and adjusted by their tutor.

However, some actions were identified to have hindered the conceptual development of empathy, and these have been highlighted as elements to be improved on. For instance, it is important to help tutors in using tutees’ prior knowledge to build new knowledge, in ensuring the effective comprehension of what has been explained, and in guiding the session. The qualitative analysis of the interaction in the final segment of the peer tutoring session (self-reflection) seems to indicate actions that must be rectified to improve the dimension referring to “Putting oneself in the patient’s shoes”. Even though the tutor involves the tutee in the activity, they do not appear to sufficiently lead the activity but instead wait for the teacher’s help. This problem has also been detected in peer tutoring practices in contexts where the teacher’s role is very relevant, and students are not used to offering pedagogical help.(11) One possible solution to help solve this problem might involve offering more autonomy to the pairs of students, which appears to be highly linked to the opportunities available to reflect on their own actions, through self-assessment. The results suggest that it is also necessary to help pairs develop quality self-assessment practices.

This study is limited by the small, non-probabilistic sample and by the specific cultural context where it was carried out. However, both limitations offer lines of future work: improving how students are assigned to intervention or comparison group, and increasing the number of students, the type of subjects and especially cultural and geographic contexts. Empathy is a cross-dimensional skill that all students from the healthcare sector should develop, as a key tool to care for patients and their families. That is why this peer tutoring programme has been created. However, not only is empathy important for nursing students, but also for any citizen in the 21^st^ century. Thus, the project reported in this article lays the foundation stone to adopt an expanded, innovative standpoint that leads to an evidence-based empathy training programme that could be adjusted and applied in any university, but also implemented in primary and secondary education.
